# Diagnostic accuracy of cardiac MRI in hemodinamically unstable patients with valvular heart disease when echocardiogram fails compared to surgical findings

**DOI:** 10.1186/1532-429X-17-S1-P227

**Published:** 2015-02-03

**Authors:** Lilia M Sierra-Galan, Begoña Parra, Alejandro Rey-Rodriguez, Maria-Elena Soto-Lopez

**Affiliations:** Cardiology, American British Cowdray Medical Center, Mexico, D.F, Mexico; Reumatologia, Instituto Nacional de Cardiologia, “Ignacio Chavez”, Mexico, Mexico

## Background

Valvular heart disease (VHD) has an incremental prevalence worldwide independent from its etiology. Currently, a variety of imaging modalities exists and are used as diagnostic tools to guide the decision between medical and surgical treatment; however, and "ideal" method does not exists and there is certain degree of discrepancy between the findings of each modality and the surgery, being this difference more dramatic in those hemodinamically unstable (HU) patients. We designed a prospective study to evaluate the diagnostic accuracy of CMR in this setting with patients usually "exclude" from this imaging modality due to the severity of its condition and their potential risks.

## Methods

We prospectively analyzed a cohort of consecutive patients with diagnosis of VHD sent to be evaluated by CMR before surgery. The IRB approved the project and all patients signed their informed consent. We include only those patients who fulfilled the inclusion criteria of being HU defined by a functional class III to IV of NYHA, Euroscore >6 points and the need of use of vasoactive agents and/or mechanical ventilation. All safety and emergency measures were taken during CMR study acquisition. An Echo was performed and reviewed within the previous 24 hours by 2 certified experts. CMR was done and interpreted by a level 3 CMR expert not blinded to Echo or clinical information and reviewed by a second expert blinded to all information. Surgery was done within the following 24 hours after CMR study. Diagnostic information of both studies was compared with the surgical findings (SF), which was considered the standard of reference. Statistical analysis was done using Spearman correlations, X^2^ and Fisher's exact test.

## Results

In a period of 18 months, a cohort of 77 patients with VHD was referred for a CMR study, of them 12 patients fulfilled the inclusion criteria, 67% were male, mean age was 55 ± 17 years old, Euroscore was 12 ± 3.7 points (range 7 - 20); 5 patients (41%) had mitral valve disease, 5 patients (42%) had aortic valve disease and 2 (17%) patients had mitral-aortic disease. The correlation between CMR and surgery was excellent with a Spearman correlation coefficient (CC) of 0.98 and kappa of 98, while the correlation between Echo and surgery was weaker with a Spearman CC of 0.35 and kappa of 0.23. Fisher's exact test showed a statistically significant difference between both imaging modalities compared with SF (p=0.001). CMR changed the previous Echo diagnosis completely in 67% of cases modifying accordingly the surgical plan. None of the patient had any adverse event inside the magnet or related to the study in the following hours before surgery.

## Conclusions

CMR is more accurate than Echo in the diagnosis of VHD in the specific subgroup of HU patients with an excellent correlation with the standard of reference. CMR guided better the surgical plan than Echo in this setting. CMR can be performed safely in this specific subgroup of patients if all safety and emergency measures are taken.

## Funding

No specific funding was used.

**Figure 1:**
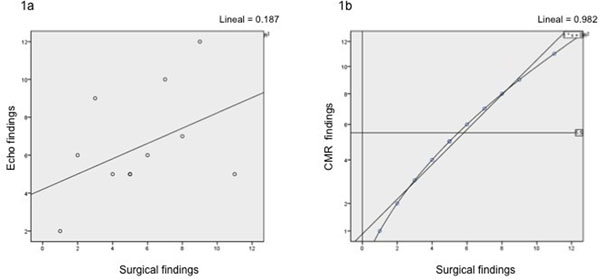
Figure 1

**Table 1:** Fisher's Exact Test for the diagnostic accuracy of the compared imaging modalities.

Imaging Modality	Accurate Diagnosis	Non Accurate Diagnosis
Echocardiogram	4	8
Cardiac Magnetic Resonance	12	0

p value = 0.001

